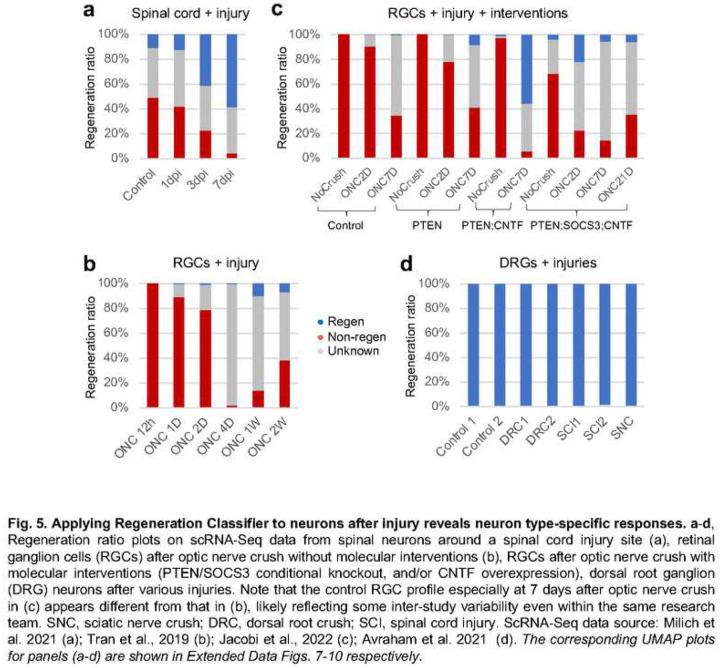# Probing regenerative heterogeneity of corticospinal neurons with scRNA-Seq

**DOI:** 10.21203/rs.3.rs-2588274/v1

**Published:** 2023-02-21

**Authors:** Hugo Kim, Junmi Saikia, Katlyn Monte, Eunmi Ha, Daniel Romaus-Sanjurjo, Joshua Sanchez, Andrea Moore, Marc Hernaiz-Llorens, Carmine Chavez-Martinez, Chimuanya Agba, Haoyue Li, Daniel Lusk, Kayla Cervantes, Binhai Zheng

**Affiliations:** University of California San Diego; University of California San Diego; University of California San Diego; University of California San Diego; University of California San Diego; University of California San Diego; University of California San Diego; University of California San Diego; University of California San Diego; University of California San Diego; University of California San Diego; University of California San Diego; University of California San Diego; University of California San Diego

## Abstract

The corticospinal tract (CST) is clinically important for the recovery of motor functions after spinal cord injury. Despite substantial progress in understanding the biology of axon regeneration in the central nervous system (CNS), our ability to promote CST regeneration remains limited. Even with molecular interventions, only a small proportion of CST axons regenerate^1^. Here we investigate this heterogeneity in the regenerative ability of corticospinal neurons following *PTEN* and *SOCS3* deletion with patch-based single cell RNA sequencing (scRNA-Seq)^2,3^, which enables deep sequencing of rare regenerating neurons. Bioinformatic analyses highlighted the importance of antioxidant response and mitochondrial biogenesis along with protein translation. Conditional gene deletion validated a role for NFE2L2 (or NRF2), a master regulator of antioxidant response, in CST regeneration. Applying Garnett^4^, a supervised classification method, to our dataset gave rise to a Regenerating Classifier (RC), which, when applied to published scRNA-Seq data, generates cell type- and developmental stage-appropriate classifications. While embryonic brain, adult dorsal root ganglion and serotonergic neurons are classified as Regenerators, most neurons from adult brain and spinal cord are classified as Non-regenerators. Adult CNS neurons partially revert to a regenerative state soon after injury, which is accelerated by molecular interventions. Our data indicate the existence of universal transcriptomic signatures underlying the regenerative abilities of vastly different neuronal populations, and further illustrate that deep sequencing of only hundreds of phenotypically identified CST neurons has the power to reveal new insights into their regenerative biology.

## Introduction

After spinal cord injury, axons from the corticospinal tract (CST) do not regenerate spontaneously to a significant extent. Extensive research has been conducted on the neuron intrinsic and extrinsic control of axon regeneration after CNS injury^5,6^. One of the first and more robust neuron-intrinsic pathways manipulated to promote regeneration is the *PTEN/mTOR* pathway: genetic knockout or shRNA knockdown of *PTEN*, a negative regulator of *mTOR* signaling, promotes CST regeneration^1,7,8^. However, even with such molecular interventions, only a few percent of CST axons regenerate, and this regeneration further declines with age^9^. Therefore, understanding the regenerative heterogeneity may be key to unlocking the mechanism of regeneration under a variety of pathophysiological conditions. In the retinal system, different retinal ganglion cell (RGC) subtypes are known to possess different regenerative capabilities and can differentially respond to molecular interventions^10,11^. Such regenerative heterogeneity has not been explored in the CST.

ScRNA-Seq is a powerful tool to dissect the molecular heterogeneity among cells. Currently, most scRNA-Seq approaches involve tissue dissociation and random barcoding, sometimes aided by fluorescence-activated cell sorting (FACS) to purify the cells of interest^12^. Single cells in suspension are then sequenced using microfluidic devices such as the Chromium controller (10x Genomics)^13^. These methods are particularly suited to profile large numbers of cells in an unbiased manner, followed by bioinformatic analyses that define cell type taxotomy^14,15^.

However, there are several drawbacks with these approaches when probing a very small, specific neuronal population with a particular phenotype, such as in the case of regenerating CST neurons. First, a very large number of cells need to be sequenced to reach a sufficient number of rare cells with the desired phenotype. With their somas residing in layer 5 of the sensorimotor cortex, CST neurons already represent a small portion of all cells through the cortical layers in the sensorimotor cortex. Assuminĝ 3% of CST neurons regenerating, we estimated that regenerating CST neurons in *PTEN*-deleted mice represent ~0.0118% of all cells in the cortical tissue harvested for scRNA-Seq [3% × 4K retrogradely labeled CST neurons / (6.8 mm^3^ × 150K cells/mm^3^)]^16^. Thus, for every 100 regenerating CST neurons profiled, one would need to sequence ~0.85 million cells, the vast majority of which would not provide directly relevant information. Second, with a relatively low sequencing depth, the 10x Genomics-based methods may not detect subtle differences (e.g., on rare transcripts) among individual neurons of the same type. Third, cell dissociation and FACS may distort the transcriptome, especially for projection neurons with long processes^17^.

These challenges can be addressed with a Patch-based scRNA-Seq method, which allows for high sequencing depth while its inherently low throughput does not present a barrier since our target neuronal population is very small. Patch-Seq was developed to capture the electrophysiological and morphological traits along with single cell transcriptomes on the same neurons, where the patch clamp pipette is repurposed to collect single cells from acute tissue slices^2,3^. Microscopy imaging can be integrated into the workflow to include morphological information, further expanding the multimodal capabilities of Patch-seq^18,19^.

To this end, we have applied Patch-based scRNA-Seq to interrogate the transcriptomic profiles of regenerating vs non-regenerating CST neurons following *PTEN* and *SOCS3* deletion. Here we primarily used Patch-Seq as a method to collect neurons under visual guidance without employing its electrophysiological capabilities. To minimize PCR biases, we adopted a recently published linear amplification method that allows for high quality, deep sequencing of high complexity transcriptomes^20^. We show that deep sequencing even on as few as hundreds of CST neurons can identify new candidates for regeneration regulators, and when analyzed in conjunction of published scRNA-Seq datasets, yields further new insights on regenerative biology.

## Results

### Experimental setup to differentially label regenerating CST neurons

We applied *PTEN* and *SOCS3* co-deletion to induce CST regeneration, as their co-deletion had previously been shown to synergistically promote axon growth (regeneration or sprouting) from RGCs and CST neurons^21,22^. To differentially label regenerating vs non-regenerating CST neurons, we applied two different retrograde viral tracers: one before and the other after a dorsal hemisection spinal cord injury (Fig. 1a). Specifically, we applied the following surgeries on *PTEN*^*fl/fl*^*;SOCS3*^*fl/fl*^*;tdTomato*^*fl/fl*^ mice along with *tdTomato*^*fl/fl*^ control mice. First, we injected AAV-retro-Cre into the thoracic cord at low T8 level at 8 weeks of age to induce *PTEN* and *SOCS3* deletion and simultaneously activate the *Rosa26-lsl-tdTomato* reporter^23^. After 4 weeks, we applied dorsal hemisection injury 500 μm above the first injection site at T8. After another 6 weeks, we injected AAV-retro-GFP at low T8 (at the original AAV-retro-Cre injection level, 500 μm below injury). We expected that, in *PTEN*^*fl/fl*^*;SOCS3*^*fl/fl*^*;tdTomato*^*fl/fl*^ mice, no more than a few percent of CST axons would regenerate ~500 μm beyond the injury site within the 10-week post-injury survival time and consequently pick up the 2nd tracer in green (GFP), whereas tdTomato would label both regenerating and non-regenerating neurons. Accordingly, green/red doubly fluorescent CST neurons would have regenerated, while red (tdTomato) only CST neurons would most likely have not regenerated (Fig. 1b,c).

Histological analyses on brain sections confirmed that GFP/tdTomato doubly fluorescent (regenerating) neurons represented only a small subpopulation of all CST neurons that were labeled in two *PTEN*^*fl/fl*^*;SOCS3*^*fl/fl*^*;tdTomato*^*fl/fl*^ mice (559/15,451 = 3.6%). No GFP labeled neurons were found in two *tdTomato*^*fl/fl*^ control mice (0/22,849 = 0%), verifying no detectable CST regeneration without molecular intervention (Fig. 1d,e).

### Patch-based single cell sequencing and differential expression analyses

We used patch pipette to collect cytoplasmic material of 326 CST neurons (123 regenerating, 203 non-regenerating) from acute brain slices of 29 *PTEN*^*fl/fl*^*;SOCS3*^*fl/fl*^*;tdTomato*^*fl/fl*^ mice. Cell collection was visually guided with both tdTomato and GFP fluorescent signals, although the setup we used did not allow for high resolution reconstruction of neuronal morphology (Fig. 1f–k). These cells were processed using a modified aRNA linear amplification protocol^20^, followed by standard Illumina TruSeq Stranded mRNA Library Prep. This method allowed us to conduct high quality, high depth sequencing of single cells. We targeted to sequence 5 million reads per cell, mapped uniquely to exons at ~1 million read pairs, which is ~100 times the depth of high quality 10x Genomics data (average 10K read pairs with sequencing saturation starting at 20K).

Because of the high sequencing depth, we analyzed our data using both bulk RNA-Seq methods (DESeq2, EdgeR) and single cell methods (Seurat, Garnett, SingleR). To this end, we first analyzed differential gene expression with DESeq2 and EdgeR. DESeq2 gave 862 differentially expressed (DE) genes, with 711 overexpressed and 151 underexpressed genes in regenerating neurons as compared with non-regenerating neurons (FDR corrected p-value < 0.05, |log2 Fold change| > 1) (Fig. 1l,m). Gene ontology (GO) analysis revealed that overexpressed genes (in regenerating neurons, as below) were enriched in ATP metabolic process (FDR = 7.51E-10), oxidative phosphorylation (FDR = 1.80E-08) and cellular respiration (FDR = 1.05E-7), indicating that mitochondrial activities are heavily involved in CST regeneration (Supplementary Table 1).

Two top differentially overexpressed genes include *ATP5A1* and *ATPIF1*, which are regulators of mitochondrial ATP synthesis, likely reflecting the requirement for high energy production during regeneration (Supplementary Table 3A, Fig. 1l). Other top differentially overexpressed genes include *SETD3*, *ATF4* and *EIF3F*. *SETD3* is an actin-specific histidine methyltransferase contributing to cytoskeleton integrity, and was recently found to mediate *PTEN* suppression-induced neuroprotection in an ischemia-reperfusion injury model by promoting actin polymerization and preserving mitochondrial function^24^. *ATF4* (Activating Transcription Factor 4) encodes a transcription factor of the cAMP response element-binding (CREB) protein family, which has another member, *ATF3*, extensively studied in peripheral axon regeneration. *EIF3F* (eukaryotic initiation factor 3F) is part of the *EIF3* complex that functions in the initiation of protein translation. Ingenuity Pathway Analysis (IPA) identified *EIF2* signaling as the top overexpressed canonical pathway (Extended Data Fig. 1), reaffirming the importance of protein synthesis. Other top pathways included oxidative phosphorylation, regulation of eIF4 and p70S6K signaling (related to mTOR and protein translation), Huntington’s disease signaling, and mitochondrial dysfunction, among others.

Through gene network analyses on all DE genes (FDR<0.05, fold change>2), we found gene hubs that control large numbers of DE genes. Two hub genes that repetitively emerged from these analyses and regulate large numbers of DE genes were *NFE2L2* and *PPARGC1A* (Fig. 1n,o). *NFE2L2* (nuclear factor erythroid-derived 2-like 2, also known as nuclear factor erythroid 2-related factor 2, or *NRF2*; not to be confused with another gene, nuclear respiratory factor 2, or *NRF-2*) encodes a transcription factor that activates antioxidant genes under oxidative stress in response to injury and inflammation^25,26^. *PPARGC1A* (peroxisome proliferator-activated receptor gamma coactivator 1-alpha, or PGC-1α) encodes a transcriptional co-activator that serves as a master regulator of mitochondrial biogenesis^27^. The Graphical Summary of IPA Core Analysis indicates that overexpression of these two genes is related to an increase in “Size of body” function, a decrease in cell death of tumor or cancer cells, and transport of molecules and vesicles (Fig. 1p). Additional hub genes include regeneration promoters *MYC* and *IGF1R*. Direct upstream regulators included *RICTOR* (Inhibited, p=1.58E-33), *MLXIPL* (activated, 2.06E-24) and *MYC* (activated, p=1.07E-21) (Supplementary Table 4). RICTOR (Rapamycin-insensitive companion of mTOR) is a component of the mTORC2 complex, which has been shown to inhibit axon regeneration^28^.

EdgeR, a weighted mean of log transformed method, detected 7751 DE genes (4813 overexpressed and 2948 underexpressed) in regenerating neurons (FDR p-value<0.05, |log2 fold change|>1) (Supplementary Table 5A). GO analysis indicates that overexpressed genes are enriched in cytoplasmic translation, various metabolic processes, ATP biosynthetic process, and oxidative phosphorylation, among others (Supplementary Table 5B), while underexpressed genes are enriched for localization functions such as cellular macromolecule localization and protein localization (Supplementary Table 5D). Of the two different models (DESeq2 and EdgeR), there was an overlapping set of 609 DE genes (474 overexpressed, 135 underexpressed in regenerating neurons, representing ~67% and ~89% of DE genes detected with DESeq2). Overlapping genes proved to be strongly significant in both EdgeR (FDR<0.01) and DESeq2 (FDR<0.001). The GO biological processes of overlapping overexpressed genes were enriched in oxidative phosphorylation and cellular respiration (Supplementary Table 6A). GO biological process of overlapping underexpressed genes were enriched in neuron differentiation and macromolecule localization (Supplementary Table 6B).

### *NFE2L2* deletion diminishes CST regeneration induced by PTEN deletion

Both *NFE2L2* and *PPARGC1A* emerged as central hubs of gene network and upstream regulators of DE genes in regenerating vs. non-regenerating CST neurons (Fig. 1n–p). We thus pursued these as top candidates of new regeneration regulators, starting with *NFE2L2*. Because *NFE2L2* was hypothesized to positively regulate regeneration, we assessed the effect of *NFE2L2* deletion in *PTEN* deletion background, which provides an elevated level of baseline regeneration so that any reduction in regeneration could be detected.

We injected AAV-Cre to the sensorimotor cortex of *NFE2L2*^fl/fl^;*PTEN*^fl/fl^ mice along with *PTEN*^fl/fl^ mice and wild-type (WT) control mice (Fig. 2a). Four weeks later, mice were subjected to dorsal hemisection spinal cord injury. Six weeks later, BDA was injected into the sensorimotor cortex to anterogradely trace CST axons, and mice were sacrificed 2 weeks later. As expected, WT mice exhibited no or little regeneration (Fig. 2b), whereas *PTEN* deleted mice exhibited significant CST regeneration (Fig. 2c)^1^. Strikingly, *PTEN* and *NFE2L2* co-deletion abrogated CST regeneration that is normally seen in *PTEN* deleted mice (Fig. 2d). We quantified CST regeneration with axon density indices rostral to injury and axon number indices caudal to injury as described^29^ (see Methods for details). This quantitative analysis verified the qualitative observation: rostral to injury, *PTEN*;*NFE2L2* doubly deleted mice exhibited a modest decrease in CST axon density indices as compared to *PTEN* deleted mice, but did not reduce to WT levels; caudal to injury, double gene deletion abolished any CST regeneration induced by *PTEN* deletion (Fig. 2e,f). RNAScope in situ hybridization confirmed a substantial reduction of *NFE2L2* mRNA levels in mouse brains (Extended Data Fig. 2). Together, these results identify NFE2L2 as a positive regulator of CST regeneration, and validate our Patch-Seq approach in discovering new regeneration regulators.

### Seurat Cluster Analysis

We next analyzed the data using single cell tools: SingleR, Seurat, Garnett. Following quality control (which removed 19 cells), we applied SingleR^30^ to transform our data into SingleCellExperiment objects, which classified the vast majority of the samples as neurons (304/307 = 99%) (Fig. 3a). Only three cells exhibited astrocyte expression profile and were excluded from further analysis.

We conducted non-supervised clustering to determine whether regenerating and non-regenerating neurons would self-segregate based on their transcriptomes. UMAP-based Seurat analysis on all data yielded two strong clusters (number of marker genes=1780, FDR<0.05). (Fig. 3b). DESeq2 analysis on Cluster 1 yielded 731 DE genes (661 overexpressed, 70 underexpressed) in regenerating neurons. While Cluster 1 is more balanced between regenerating and non-regenerating neuron, Cluster 2 was enriched in non-regenerating neurons (43 non-regenerating, 10 regenerating) (Fig. 3c). There were no significant DE genes between regenerating and non-regenerating neurons within Cluster 2.

### Building Regeneration Classifier with Garnett

Unsupervised clustering above may not capture all transcriptomic features likely due to both the relatively low sample size coupled with the unusually high sequencing depth^31^. To gain further insights, we turned to an R package called Garnett, a supervised clustering tool to generate custom cell type classifier based on scRNA-Seq data^4^. Using both clusters found from Seurat and the regeneration phenotype within Cluster 1, we trained the program to specifically detect regenerating CST neurons. Based on the p-value and logFC of markers found from DE genes in each cluster and between regenerating and non-regenerating neurons, we generated the marker file. Using the marker file (initial marker genes listed in Fig. 3d) and scRNA-Seq data, we trained the program to generate the Regeneration Classifier (RC), which includes 4 groups: Cluster 1 regenerating, non-regenerating, and unknown; Cluster 2 unknown (marked as Cluster 2) (Fig. 3c).

We applied the RC to gauge the regenerative ability of neurons based on published scRNA-Seq data. First, we applied it to a recent adult mouse primary motor cortex dataset (10x v3) as this represents the cognate anatomical site for CST neurons^15^. To our initial surprise, a substantial portion of glutamatergic neurons (which include CST neurons) were classified as regenerators (Fig. 3e,f). Restricting the analysis to glutamatergic neurons revealed cortical layer and neuronal projection-specific classifications (Fig. 3g,h). While most of extratelencephalically projecting neurons were non-regenerators, L6 intratelencephalically projecting neurons and L6b neurons were mostly regenerators (Fig. 3i). L5 intratelencephalically projecting neurons were a mixed population. These observations are consistent with a previous *in vivo* imaging study indicating that L6 neurons exhibit a higher frequency of regeneration than L2/3/5 following laser axotomy^32^. Applying RC to scRNA-Seq data from adult raphe nucleus^33^ and DRG neurons^34^ classified these two neuronal populations mostly as regenerators (raphe: 96%; DRG: 87–99%) (Fig. 3j), consistent with the observations in the literature that 5HT raphe neurons and DRG neurons tend to regenerate better following CNS injury^35,36^.

### Regeneration Classifier reflects neurodevelopment stage

One prediction for a valid RC would be that it would follow a developmental timeline, which may vary among different neuronal types. We applied the RC to published scRNA-Seq dataset from a list of neuronal types or CNS regions across developmental stages (Supplementary Table 9, Extended Figs. 3–6, Supplementary Figs. 1–3). Each dataset was analyzed for all cell types as well as neurons only. Based on the result, we measured the regenerating ratio defined as regenerating neuron numbers over sum of regenerating and non-regenerating neurons (assuming unknowns have the same ratio of regenerating vs non-regenerating neurons).

Results indicate that many neuronal types and CNS regions lose their regenerative potential between birth and postnatal day 23, roughly corresponding to the juvenile stage (Fig. 4) (also see Supplementary Information for more detailed results). Neurons in the ventral midbrain exhibit an earlier partial decline by around E15.5. RGCs exhibit a sharp drop of regenerative potential between birth and P5, strikingly resembling the pattern from previous *in vitro* studies^37^ (Fig. 4; Extended Data Fig. 3, compare panels *g* and *h*). Sensory cortex, prefrontal cortex, hypothalamus, visual cortex, and motor cortex in turn lose their regenerative potential between P17–21 and P56 (Extended Data Fig. 4, 5). Motor cortex did not completely lose its regenerative potential by P56. Spinal cord neurons exhibit an incomplete loss of their regenerative potential by P56, again likely reflecting spontaneous regenerative ability of some spinal interneurons^38^ (Extended Data Fig. 6). Cerebellum did not lose most of its regenerative potential by P17–21 (which lacks data for P28–56), while DRG and raphe nuclei retain most of their regenerative potential by P56 (the latest time point analyzed). These results illustrate that our RC has predictive value to gauge the regenerative potential across neuronal types, anatomical regions, and developmental timeline. Future studies will reduce noise and refine its predictive power.

### Applying Regeneration Classifier to other axon injury studies

Applying the RC to scRNA-Seq data from published CNS injury studies revealed a partial transition from non-regenerators to regenerators that is accelerated by molecular interventions (Fig. 5). Adult spinal neurons exhibit a notable baseline regenerative potential^39^, likely reflecting that of some interneurons (Extended Data Fig. 7)^38^. Following spinal cord injury, a gradual transition occurred from non-regenerators to regenerators within the first 7 days post injury (Fig. 5a). In the retinal system, adult RGCs exhibited a very low baseline regenerative potential, yet the same transition towards a high regenerative potential occurred within 7 days after optic nerve crush (Fig. 5b, Extended Data Fig. 8)^40^. These data are consistent with a previous study indicating that neurons revert to a transcriptional growth state soon after injury without any molecular or cellular intervention^41^. With molecular interventions such single, double or triple gene manipulations (involving *PTEN;SOCS3* loss of function and/or *CNTF* gain of function), this reversion is accelerated in the retinal system (Fig. 5c, Extended Data Fig. 9)^42^. Applying the RC to DRG neuron data with multiple injury models^43,44^ revealed a high regenerative potential for pre-injury DRG neurons that did not substantially change after injury (Fig. 5d, Extended Data Fig. 10). While some of the unknown classification may be due to insufficient transcriptomic information, the temporal pattern of transition suggests that the unknown classification may also reflect a transitional state between regenerators and non-regenerators.

## Discussion

In this study, we adapted a Patch-seq workflow to conduct single cell sequencing on regenerating CST neurons following *PTEN*;*SOCS3* gene deletion. The high sequencing depth afforded by this approach allowed us to conduct bulk-seq analyses on differential gene expression in addition to single cell analyses such as Seurat and Garnett. The former led to the identification of new candidates of regeneration regulators, one of which, *NFE2L2* (also known as *NRF2*), has been validated with *in vivo* injury models. The latter led to the development of a Regeneration Classifier that exhibits predictive value for the regenerative abilities of a wide spectrum of neuronal types based on their single cell profiles. Thus, deep sequencing of even hundreds of neurons may reveal new biological insights into neuronal regeneration following traumatic injuries.

The GO and IPA analyses illustrated the critical importance of protein translation, oxidative stress response, and mitochondria biogenesis/function in CST regeneration. Regenerating CST neurons differentially overexpress genes and pathways involved in protein translation including eIF2, eIF3F, eIF4 and p70S6K. This is in line with the published literature on PTEN/mTOR and other pathways demonstrating the importance of protein synthesis in CNS axon regeneration^1,45^. In our study, both regenerating and non-regenerating CST neurons underwent *PTEN;SOCS3* gene deletion and hence their transcriptomic differences do not necessarily reflect differences in PTEN/mTOR or SOCS3/STAT3 signaling. Other genes/pathways known to regulate axon growth and regeneration were also captured in our study, such as MYC, IGF1R and HTT (Supplementary Table 4). MYC overexpression synergizes with *PTEN;SOCS3;CNTF* manipulations to promote robust retinal axon regeneration after optic nerve crush^46^. Administration of IGF1R antibodies blocks CST axon extension in the postnatal spinal cord^47^. HTT (Huntingtin) mediates host CST axon regeneration into neural stem cell graft implanted at a spinal cord injury site^41^.

Compared with protein translation, much less is known about the role of antioxidant response and mitochondria biogenesis in CST regeneration. In our study, *NFE2L2* and *PPARGC1* emerged as two top upstream regulators of pathways enriched in regenerating neurons. NFE2L2 (NRF2) is a master regulator of the antioxidant defense system^48^. Upon oxidative stress, NRF2 enters the nucleus and mediates the transcription of antioxidant genes through binding to an enhancer element called antioxidant response element (ARE). Extensive studies have shown a cytoprotective role for NRF2 in a variety of pathological conditions including inflammation, cancer, cardiovascular and neurodegenerative diseases. *NFE2L2;PTEN* double gene deletion abolishes CST regeneration induced by *PTEN* deletion, pointing to an important role for NRF2-mediated antioxidant response in CST regeneration. As aging reduces the expression level of NRF2^49^, this may partially account for the age-dependent decline in CST regeneration previously reported with PTEN deletion^9^. Conversely, a previous study indicates that reactive oxygen species (ROS) is required for peripheral and dorsal column sensory axon regeneration^50^. Thus, the complex roles of ROS, oxidative stress and antioxidant defense system, including any neuronal type-specific regulation, remain to be fully elucidated.

Another top candidate, PPARGC1A (or PGC-1α), is a master regulator of mitochondrial biogenesis^51^. Although the *in vivo* role of PGC-1α in CST regeneration remains to be validated, our observation that these two genes (*NFE2L2, PPARGC1A*) sit at the top of the regulatory network in regenerating neurons highlights the critical importance of both antioxidative response and mitochondria biogenesis. Indeed, mitochondrial function and dysfunction are intimately linked to oxidative stress and redox state within the cells. There is evidence that *NFE2L2* cross-regulates with genes involved in mitochondrial biogenesis and function^52^. Conversely, PGC-1α has a role in antioxidant response^53^, and can co-activate the transcription of *NFE2L2*^26^; PGC-1α and NFE2L2 may even cross regulate^54^. Thus, resolving oxidative stress and maintaining healthy mitochondria function are likely two important and related aspects of CST axon regeneration. Previous studies stressed the importance of mitochondrial motility and energy metabolism in CNS regeneration^55–57^. The current study emphasizes the importance of countering the negative consequences of mitochondrial dysfunction. Outside of NFE2L2 and PGC-1α, other top candidates such as SETD3, if functionally validated *in vivo*, may lead to additional biology insights.

We used the Garnett R package to train a Regeneration Classifier based on DE genes within Cluster 1. We found that our Regenerating Classifier can be applied in an unbiased manner to characterize any published single cell dataset. This generated a pattern of regeneration classification for various neuronal populations that remarkably mirrors prior knowledge on their regenerative potential based on the neuronal type and developmental stage. Overall, embryonic neurons tend to be classified as regenerators while adult neurons exhibit the opposite trend. Within adult neurons, while RGCs and many other CNS neuronal types exhibit a low regenerative potential, DRG and serotonergic neurons in the raphe nuclei exhibit a high regenerative potential based on the RC, with other neuronal types falling in between. Applying the RC to CNS injury datasets (CST neurons and RGCs) corroborated with an emerging hypothesis that CNS neurons revert to a regenerative state after injury^41^, which can then be accelerated (and possibly sustained) with molecular intervention. In contrast, DRG neurons sustain a high regenerative potential after sciatic nerve crush, dorsal root crush or spinal cord injury, indicating the involvement of neuron-extrinsic influences in the different regenerative outcomes following different types of injury. Because our Regenerative Classifier was developed based solely on data from CST neurons following *PTEN;SOCS3* deletion, these results also indicate the existence of some universal transcriptomic features underlying the regenerative abilities of many different neuronal populations.

Finally, our work highlights the value of deep sequencing on a relatively small number of neurons in studying the biology of CNS axon regeneration. As such, deep sequencing of even hundreds of CST neurons may lead to the identification of new regeneration regulators and the development of a widely applicable Regeneration Classifier. A recent study applying high depth SMART-Seq2 to hundreds of FACS-purified RGCs demonstrated that high depth, low sample size sequencing can distinguish two rare, transcriptionally similar neuronal subtypes that could not be distinguished with droplet-based scRNA-Seq^58^. Therefore, high depth, low throughput scRNA-Seq methods have a unique niche in distinguishing transcriptionally similar neuronal subtypes or even states. In our study, the differential gene expression between regenerating and non-regenerating CST neurons likely reflected the different regenerative states rather than neuronal subtypes. High depth, low throughput scRNA-Seq methods will continue to complement low depth, high throughput methods in understanding new biology.

## METHODS

### Mice

We used *PTEN*^*fl/fl*^*;SOCS3*^*fl/fl*^*;tdTomato*^*fl/fl*^ mice for Patch-Seq experiments and *tdTomtato*^*fl/fl*^ mice for control. Cre mediated recombination induces gene deletion for *PTEN* and *SOCS3*, and simultaneously activates the *tdTomato* reporter gene targeted to the ROSA26 locus. For *NFE2L2* function validation experiment using genetic loss of function, we used *NFE2L2*^*fl/fl*^ mice from Jackson Laboratory (C57BL6-*Nfe2l2*^*tm1.1Sred*^/SbisJ ,Strain #: 025433) and bred this line to *PTEN*^*fl/fl*^ mice to obtain *NFE2L2*^*fl/fl*^*;PTEN*^*fl/fl*^ mice. All mice were assessed in C57BL/6 background. All procedures were approved by the Institutional Animals Care and Use Committee at University of California San Diego and at VA San Diego.

### Mouse surgeries

We performed three mouse surgeries prior to Patch-Seq based cell collection. At the age of 8 weeks, AAV-retro-Cre (10^13 gc/ml titer, Boston Children’s Hospital Viral core; same below) was injected to low T8 level of the spinal cord. Using a 10 μl Hamilton Syringe with a glass pipette attachment, we injected 0.8 μl virus at 0.1 μl/min to 0.5 mm left from the center of the spinal cord and 0.5 mm deep unilaterally. Four weeks after the first injection, dorsal hemisection (0.7 mm depth) was performed 0.5 mm above the injection site at T8, as previously described to lesion the main and dorsolateral CST axons^1,2^. A pair of superfine straight vannas scissors (Cat # 501778, WPI) were used to cut the dorsal half of the spinal cord in multiple cuts at 0.7 mm depth, and the injury completeness was ensuring by passing a microfeather blade at 0.7 mm depth (Cat # 200300715, pfm medical) once in both directions. After 6 weeks, AAV-retro-GFP (10^13 gc/ml titer) was injected at the same injection site for the first injection. Four weeks after the second injection, we sacrificed the mice and collected single cells using a patch clamp setup. A small number of mice underwent the same procedures but were perfused with 4% Paraformaldehyde (PFA) instead for immunohistochemical examination and quantification of retrogradely labeled CST neurons.

For *NFE2L2* function validation experiment, we performed three mouse surgeries prior to the terminal procedures. At the age of 8 weeks, we injected AAV-Cre (10^13 gc/ml titer) Virus at a rate of 0.1 μl/min for 4 min, total 0.4 μl per injection unilaterally at 0.5mm depth to the hindlimb-projecting sensorimotor cortex after craniotomy (Coordinate: 1.4 mm lateral, 0.1 mm posterior; 1.0 mm lateral, 0.6 mm posterior; and 1.4 mm lateral, 1.1 mm posterior from bregma) as previously described^1,2^ After 4 weeks, we perform the dorsal hemisection spinal cord injury at T8. After 6 weeks, biotinylated dextran amine (BDA, Cat # D1956, Invitrogen) was injected into the same coordinates of the cortex as AAV-Cre above to trace CST axons. Mice were sacrificed 2 weeks after the BDA injection with pentobarbital followed by PFA perfusion.

### Immunostaining and microscopy imaging

For quantification of retrogradely labeled CST neurons, DAPI (1:5000) was applied to stain the nuclei and sections were mounted on Superfrost Plus slides and Fluoromount-G was used as the mounting medium. The slides were visualized under a Zeiss AxioImager M1 fluorescence microscope using separate filters for GFP, tdTomato and DAPI. GFP and tdTomato were visualized with their native signals without immunostaining. Images were taken and the numbers of neurons carrying GFP and/or tdTomato signals were quantified.

For *NFE2L2* function validation experiment, sagittal spinal cord and transverse medulla sections were stained with rat anti-GFAP primary antibody (Cat # 13–0300, ThermoFisher) and anti-Rat secondary antibody conjugated with Alexa 488 (Cat # A-11006, ThermoFisher). BDA was stained with ABC kit (PK-4000, Vector Labs) and TSA reagent Cy3.5 (NEL744001KT, Perkin Elmer), as described^2^. Tissue sections were imaged with 10x and 20x objectives. To assess CST regeneration, we quantified the rostral axon density indices and the caudal axon number indices as previously described^2,3^. Specifically, labeled CST axon densities measured at defined distances rostral to the injury site were averaged over ~10 sagittal sections and then normalized against the axon density at 1.5 mm rostral to injury; labeled CST axon numbers counted at defined distances caudal to the injury site were averaged over ~10 sagittal sections and then normalized total axon count in the medulla. All quantifications were conducted by experimenters blinded to the genotypes, sometimes by multiple experimenters independently to check the results.

### RNAScope

To confirm conditional *NFE2L2* gene deletion in brain tissue, a custom fluorescent RNAScope Multiplex Fluorescent V2 Assay (ACD Biotechne, Cat #323100) was used to detect mRNA. Probes targeting *NFE2L2* (exon 5) and *Bcl11b* (marker for CST neurons) were used. For each experimental or control condition, coronal brain sections measuring 20 μm in thickness from three mice per genotype were selected spanning the region of AAV-Cre injections. The sections were mounted and baked onto Superfrost Plus slides (Thermo Fisher Scientific) and blocked for endogenous peroxidase activity; subsequently slides underwent antigen retrieval treatment and allowed to dry overnight. On the following day, sections underwent protease treatment, and probes were hybridized and amplified. Signal was detected with TSA Vivid^™^ Fluorophore Kit 570 (Tocris, Cat #7526) and TSA Vivid^™^ Fluorophore Kit 520 (Tocris, Cat #7523), and counterstained with DAPI for nuclei; 10× images were taken spanning the entire cerebral cortex, and 20× images were taken at Layer V.

### Patch clamping and single cell extraction

Mice were sacrificed with Ketamine/Xylazine mix followed by perfusion with bubbling sucrose cutting solution and decapitation. Mouse brains were sliced with VT1000 vibratome (Leica) into 200–400 μm slices. Neurons in acute brain slices were visually identified under illumination with an infrared Dodt Gradient Contrast system (Scientifica) and fluorescence (488 and 594 nm). Patch pipettes (6–10 MOhm; 1.2 mm O.D.) were filled with intracellular solution (K-gluconate 130 mM; KCl 2 mM; CaCl_2_ 1 mM; MgATP 4 mM; GTP 0.3 mM; phosphocreatine 8 mM; HEPES 10 mM; EGTA 11 mM; pH 7.25 and 300 mOsm) containing 0.4 U/μl recombinant RNase inhibitor (Clontech).

Under the Dot contrast, the suction pipette was used to remove the top layer of the tissue slice including dead debris and connective tissue to expose CST neurons. Then, the patch pipette tip was lowered. Using red fluorescence (tdTomato), CST neurons were identified, and patch pipette was approached to the cell to form a giga seal. After making whole cell configuration, using the strong attachment, the entire cell on patch pipet was lifted straight out of the solution. The content was expelled into a PCR tube containing 5 μl of lysis buffer (made using NaCl 350 mg, Triton 500 μl, NP-40 500 μl, deoxy 2.5 ml, Tris HCl pH 8.8 1 ml, Tris HCl pH 6.8 1.5 ml, HEPES 240 mg, pH adjusted to 8) by breaking the end of pipette tip and immediately flash frozen using liquid nitrogen. The cellular material was centrifuged followed by 1 to 2 freeze thaw cycles to ensure complete lysis of the material.

### Modified aRNA protocol

We processed collected single cells using the modified aRNA protocol previously described^4^ followed by Illumina TruSeq Stranded mRNA library prep (Cat # 20020594, Illumina).

### Sequencing and data processing

Size distribution of sequencing libraries was assessed by Agilent D1000 Screen Tape (5067–5582) on an Agilent 2200 TapeStation, and library concentrations were measured by Qubit from the IGM Genomics Center at UC San Diego. Libraries were multiplexed and sequenced on an Illumina NovaSeq System with 150 bp pair-ended reads and trimmed to 100 bp reads.

Sequencing reads were further trimmed from both ends based on quality score. The trimmed reads were mapped to mouse genome (Release M22, GENCODE) using STAR aligner (STAR - 2.5.3a) at the Triton Shared Computing Cluster (TSCC), UC San Diego^5^. Mapped samples were processed using HTSeq and made into read tables.

### Data analysis and developing Regeneration Classifier (RC)

Because our Patch-Seq data with linear amplification is different from regular 10x based single call data or bulk RNA, we used both DESeq2 and EdgeR to identify differentially expressed (DE) genes. We conducted Gene Ontology (GO) analysis using the gene list and p-values from our data using topGO package. For clustering, we generated a Seurat object from the data and ran through the Seurat workflow. Cell types were initially classified using SingleR package. Upon publication, we will share our code in the following GitHub page: https://github.com/neurohugo/SingleCellPatchseqAnalysis.

The Ingenuity Pathway Analysis (IPA) was done with two different methods. First, for the independent network analysis, we applied all DE genes to the field and connected them together. Then we applied the Grow function to expand the network, limited only one step upstream. We removed all the unconnected genes and organized hierarchically to find the hub genes. We removed the non-hub upstream genes for Fig. 1n. In addition, we independently applied the Core Analysis and Graphical Summary.

Finally, we generated the Regeneration Classifier (RC) from the DE genes in regenerating neurons of Cluster 1 using Garnett^6^. We used Garnett to train cell type classifier using the dataset we obtained in this study, and applied it to other datasets from the scRNAseq package (Bioconductor)^7^ and published papers (listed in Supplementary Table 9). All data were transformed into Seurat objects^8^ and classified using the Regeneration Classifier. Using the RC, we also generated the package “RegenOrNoRegen”, which is available in the same GitHub page.

### Data repository

Single cell sequencing data is available in GSE205769. The package for RC application is available in the GitHub page above.

## Figures and Tables

**Figure:**
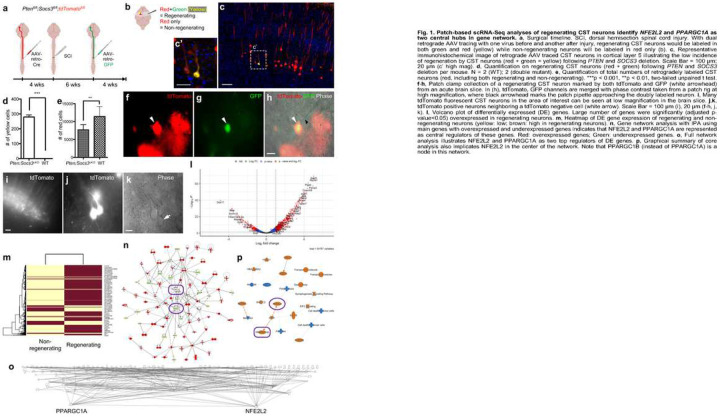


**Figure:**
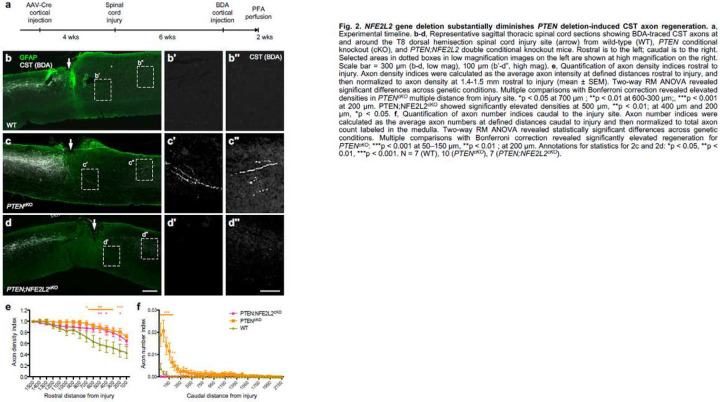


**Figure:**
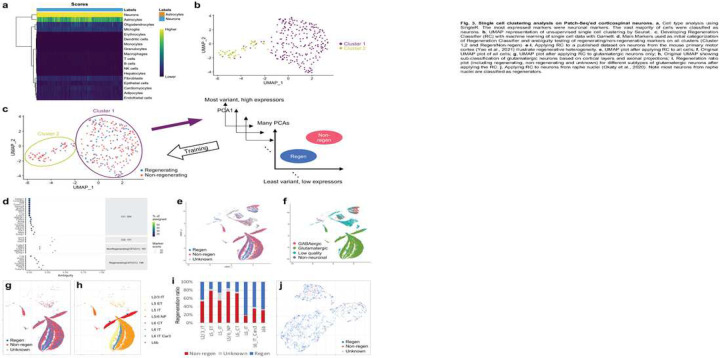


**Figure:**
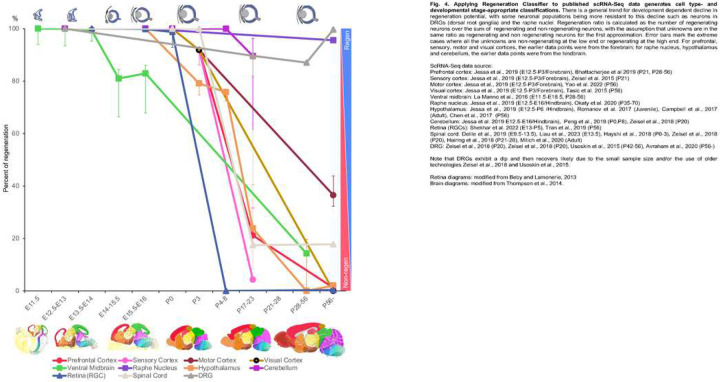


**Figure:**